# Preoperative skull tongs-femoral traction versus cotrel longitudinal traction for rigid and severe scoliosis: Cohort study

**DOI:** 10.1016/j.amsu.2021.02.023

**Published:** 2021-02-12

**Authors:** Didik Librianto, Reza Saputra, Yoshi Pratama Djaja, Phedy Phedy, Fachrisal, Ifran Saleh

**Affiliations:** a Department of Orthopedic & Traumatology, Fatmawati General Hospital, Jakarta, Indonesia; b Department of Orthopaedic & Traumatology, Cipto Mangunkusumo General Hospital, Faculty of Medicine Universitas Indonesia, Jakarta, Indonesia

**Keywords:** Severe scoliosis, Skull tong femoral traction, Cotrel traction, Preoperative traction, Scoliosis

## Abstract

**Background:**

To compare two methods of preoperative traction (Cotrel traction exercises and skull tongs femoral traction) in severe scoliosis treatment.

**Methods:**

We collected retrospective data of severe (>80°) and rigid scoliosis patients who underwent preoperative traction before correction surgery from 2016 to 2018. The first group consisted of patients who underwent Cotrel traction exercises and second group underwent continuous-progressively increasing Skull Tongs Femoral Traction (STFT) traction. Posterior fusion was performed in all patients. Intraoperative parameters (blood loss, operation time and level instrumented) and radiologic change (initial, post-traction and postoperative Cobb Angle) was evaluated and analyzed.

**Results:**

Thirty consecutive case of severe and rigid scoliosis were included (15 in each group). Despite Cotrel group having larger initial Cobb angle, the amount of post traction correction was statistically similar in both groups (16.4**°** and 11.8**°**, in STFT and Cotrel group respectively). Mean traction duration was 14.0 days for Cotrel group and 12 days for STFT. There were also no significant differences in postoperative curve correction rate between two groups, although STFT group had a slightly higher correction rate (69.3**°** vs 55.0**°**). No major/neurologic complication were found in our series.

**Conclusions:**

Both preoperative traction methods were found safe and beneficial to reduce preoperative curve degree before definitive scoliosis correction surgery. Although, no statistical difference were found between two methods, STFT may provide better correction rate.

**Level of evidence:**

3.

## Highlights

• Compare two preoperative traction methods in severe scoliosis.
• Preoperative traction were safe and beneficial to reduce preoperative curve degree.
• Skull Tongs Femoral Traction may provide better correction rate.

## 1 Introduction

Scoliosis is a complex three-dimensional deformity of the spine which characterized by a combination of lateral curvature and rotation of the vertebrae [1,2]. The degree of the scoliosis curvature is associated with its severity and in severe scoliosis, the acute correction is not only challenging but also poses a high risk of complications [1,3]. Several methods have been demonstrated to obtain an optimal correction for severe scoliosis: anterior release, spinal osteotomies, apical vertebrae resection, temporary internal distraction, perioperative traction and some other additional procedures [4].

External distraction is not an uncommon method for scoliosis correction. It can be performed preoperatively, during the surgery or postoperatively after an initial release procedure. In severe spine deformity, the use preoperative spinal traction (halo-femoral traction, halo-gravity traction, etc.) has been demonstrated to improve curve flexibility [4]. Since the patient was awake during the traction, neurologic function can be closely monitored in order to reduce the risk of permanent neurologic deficits [3,5].

The development of halo vest traction that was initiated by Nickel and Vernon in 1950s marked an important milestone for the treatment of severe spinal deformity. After that, halo-gravity traction and halo-femoral traction had been introduced as a safe and effective method for treating spinal deformities [6,7]. Although biomechanically inferior than halo, skull tongs has also been used as an alternative in preoperative traction in scoliosis [8,9].

Other preoperative traction method is Cotrel longitudinal traction, which was introduced initially as a conservative method for treating adolescensce idiopathic scoliosis [10,11]. It is less invasive than halo/skull tongs traction thus have a lower complication rate. Several studies stated that it was ineffective as a nonoperative treatment for scoliosis [12]. However, its effectivity as preoperative traction for spine deformity is still questionable [11]. It is hypothesized that the dynamic exercise in Cotrel traction, may improve curve flexibility despite providing lesser traction forces than halo-femoral traction. An option of less invasive distraction method is always favorable. Therefore the purpose of present study were: (1) determine efficacy of skull tongs-femoral tractions (STFT) and Cotrel longitudinal tractions, (2) compare and define the better preoperative traction protocol for severe scoliosis, and (3) evaluate the safety/incidence of complication.

## 2 Materials and methods

Institutional review board approval was obtained and the need for informed consent was waived due to the retrospective nature of the study. The study was performed in line with the STROCSS criteria [13] and registered in clinicaltrials.gov with identification number of NCT04671147 (https://clinicaltrials.gov/ct2/show/NCT04671147). A total of 30 consecutive patients with severe and rigid scoliosis who underwent preoperative traction before correction surgery were recruited for this study. All surgeries were performed by one or two surgeons in a single tertiary spine center hospital between 2016 and 2018. Inclusion criteria were: patients aged 10–40 years old at the time of surgery; adolescence/adult idiopathic scoliosis; severe and rigid scoliosis (defined as Cobb angle more than 80° and flexibility index less than 25%) [9]; either skull tongs-femoral traction or Cotrel longitudinal traction used in the preoperative time period.

Patients who was diagnosed with other types of scoliosis (neuromuscular scoliosis, congenital scoliosis, etc.) were excluded. Patients that had intradural abnormalities (diastomatomyelia, tethered cord, etc) or history of previous spine surgery were also excluded.

Chart review was performed to analyze the patients demographic at the initial examination (age, gender and BMI), major coronal curve magnitude, major compensatory coronal curve magnitude, major sagittal curve magnitude, flexibility index, traction protocol and surgical procedure performed. Short and long term complication were noted in each case.

Standing anteroposterior and lateral spine radiograph were within 2 months before the traction. All radiograph should expand from C7 to S1 vertebrae. In each case, a follow-up supine anteroposterior spine radiograph were taken weekly while patients were in traction. After the traction protocol, immediate long standing anteroposterior and lateral spine radiograph were taken and reviewed, especially the coronal and sagittal curve magnitude change.

**Skull Tongs-Femoral Traction (STFT) Protocol** consists of rigid semi-circular bar that follows the coronal contour of the head with a hole on each end that allows the placement of a pin into the outer table of the temporal region. The pins trajectories are directed in the vector of the pull to minimize pullout during traction [14].

**Fig. 1:**
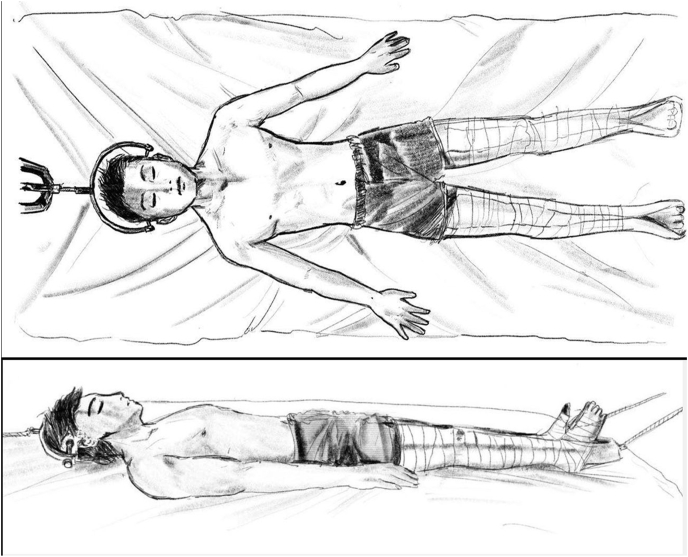
Illustration for skull-tong femoral traction.

The skull-tongs were applied under local anesthesia. An initial traction was performed by giving 2.5–5 kg weight, which was increased for 1 kg a day. It was continued until target Cobb angle of 60° or maximum load of 40% body weight was obtained. The head of the bed were elevated about 5–10 cm (as needed) to provide gravity aids (Fig. 1). After the target was achieved, the traction was maintained 2–3 days and followed by surgical correction. The traction was maintained throughout the surgery however; the weight was decreased by 50%.

### 2.1 Cotrel dynamic traction protocol

**Fig. 2:**
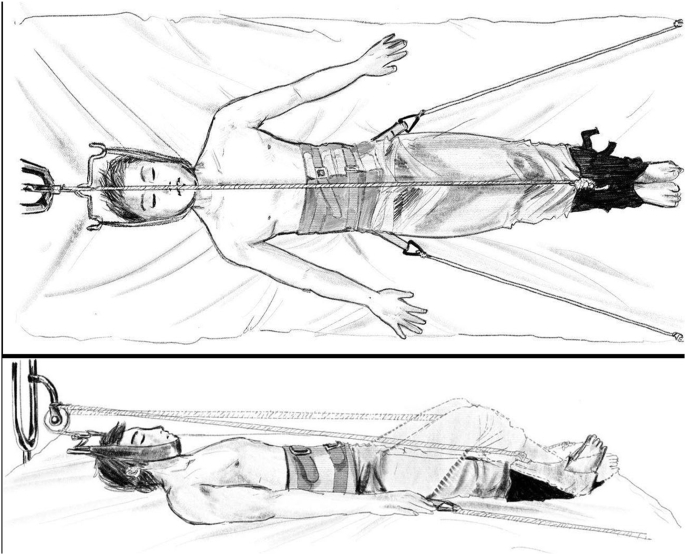
Illustration for cotrel traction.

Cotrel traction has three major components: (1) head halter with occipital piece and a chinstrap that attached to an adjustable weight by rope and a spreader bar; (2) pelvic straps system to counterbalance the pulling force on the head halter; (3) two foot-pedals and ropes system that provide dynamic traction to the spine [11]. (Fig. 2)

After the Cotrel system had been installed, a continuous head-pelvic traction by 2–3 kg static weight was applied. The static traction was provided by the head halter and pelvic straps system. Forty-five degree angle of pull between horizontal plane and the head halter's rope were maintained to place the pulling force on the occiput rather than the chin. The foot of the bed was also elevated for 5–10 cm to provide gravity aids and preventing the pelvic strap from becoming slack.

The patient was also educated to perform intermittent exercise by extending his/her knees with the foot-pedals on at least 10 min in every hour during daytime. The amount of force given were based on the patient's own tolerance. The patients were ambulatory for about 2 h every day throughout the traction period for personal care. The traction was maintained until 2–3 weeks and was released before the surgery.

### 2.2 Surgical correction & follow up

All patients were operated by one senior author (DL) and assisted by one of two other authors (FC and PH). Under general anesthesia and the control of intraoperative monitoring, all patients underwent posterior spinal instrumentation. Medial facetectomy was performed to provide some mobility to the spine. Additional Ponte osteotomy was performed if needed. Spinal deformity was corrected through derotation technique using prebent titanium rod. Posterior fusion was accomplished using autogenous local bone graft and synthetic bone graft.

Intraoperative parameters such as operation time, blood loss, and level instrumented were measured and recorded. Patient were closely monitored during the postoperative course especially the neurologic status. Post operative radiograph were obtained as soon as patient's condition allowed, from which the post-operative coronal and sagittal curve magnitude, post-operative correction rate (post-traction and after surgery) and total correction rate (pre-traction and after surgery) were measured. Patients were followed until 1 year after surgery. Any complication found from the traction period until the final follow up were recorded and treated accordingly. Major complication included neurologic injury, pin penetration, osteomyelitis, and subdural abcess. Minor complication included pin loosening, localized infection, periorbital edema, superficial pressure sores, and unsightly scars.

The chi-square test was used to analyze categorical variables. The independent *t*-test and Mann-Whitney test were used to analyze continuous variables. All reported p-values are two sided, and p-values of <0.05 were deemed significant. Data were analyzed using SPSS 23.0 (SPSS, Chicago, Illinois).

## 3 Results

**Fig. 3:**
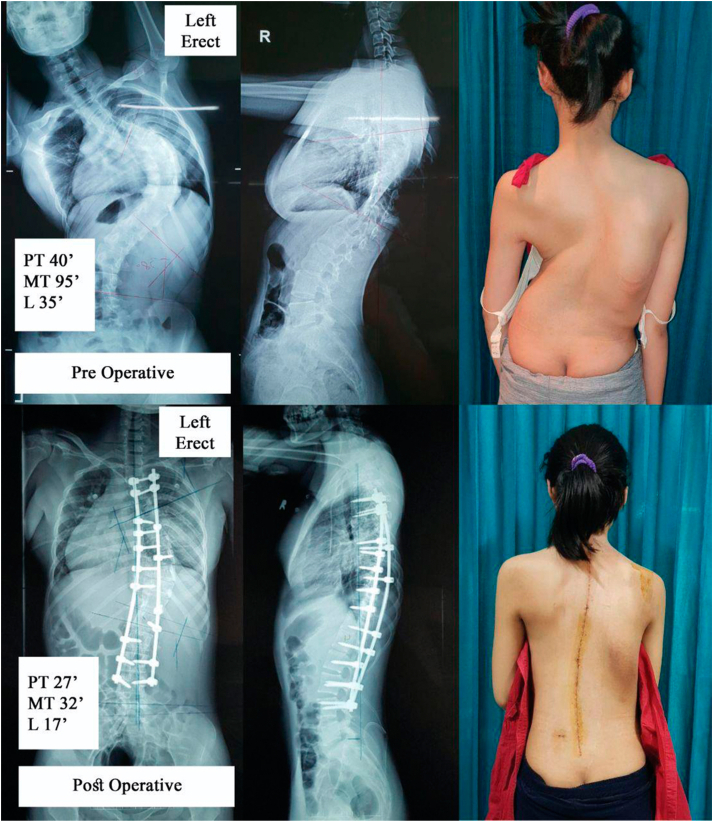
Female 20 years old treated with preoperative skull tong femoral traction. Initial Cobb angle was 95**°,** which was corrected to 32**°** (Main thoracic curve).

**Fig. 4:**
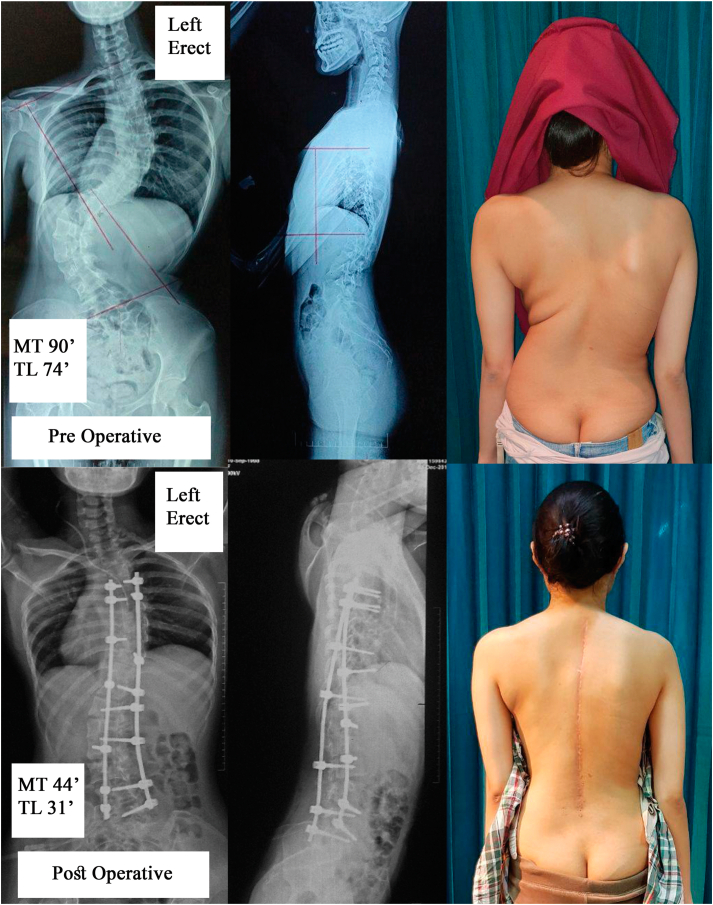
Female18 years old treated with preoperative Cotrel longitudinal traction. Initial Cobb angle was 90**°**, which was corrected to 44**°** post operatively.

Twenty patients were included in the analysis. The average duration of traction was 12.4 days (range, 7–21 days). All patients underwent definivite posterior spinal fusion through single posterior approach (Fig. 3, Fig. 4). One patient from STFT group underwent Ponte osteotomy.

Demographic, traction and operative variables are listed in Table 1.

**Table 1:** Patients demographic.

	GWT	Cotrel	p value
Number of patients	15	15	
Age	20.6 ± 2.9	20.4 ± 2.3	
Gender (M/F)	5/10	4/11	
BMI	22.9 ± 4.3	23.5 ± 4.1	
Duration of traction (days)	14 (7–21)	11 (7–17)	0.008
Maximum traction weight (kg)	7 kg	3 kg	
**Curves**			
Major coronal curve	100.9^o^ ± 7.6^o^	102.3^o^ ± 11.2^o^	0.004
Compensatory coronal curve	34.3^o^ ± 7.3^o^	37.5^o^ ± 14.6^o^	
Flexibility index	9% (4–22)	8% (3–14)	0.01
Sagittal plane curve	36.5^o^ (13° – 54°)	40.0° (10° – 80°)	
**Operative Parameter**			
Operation time (minutes)	240 (180–360)	225 (180–300)	0.541
Blood loss (mL)	1530 ± 573.5	1610 ± 696.7	
Level fused	14 ± 1.7	12.2 ± 2.5	
Spinal osteotomy needed	1/10	0/10	

Although it is statistically insignificant, the Cotrel group have a higher average initial major coronal curve magnitude than the STFT group as seen in Table 2. Patients who underwent STFT obtained better correction rate post traction and post surgical correction. The surgical correction rate was comparable between two groups.

**Table 2:** Main coronal curve comparison.

	Skull-Tongs Femoral Traction	Cotrel Traction	p Value
**Initial Cobb (°)**	100.9 ± 7.6	102.3 ± 11.2	0.623
**Post Traction Cobb (°)**	84.5 ± 13.9	90.5 ± 10.0	0.560
Traction Corr. Rate* (%)	16.3 (3.3–40.8)	10.4 (2.9–22.2)	0.187
**Post Operative Cobb (°)**	31.0 (24.0–34.0)	46.0 (4.0–55.0)	0.098
Surgical Corr. Rate** (%)	53 (18.4–73.08)	44.6 (22.3–88.0)	0.108
Total Correction Rate*** (%)	69.3 ± 3.9	55.0 (41.4–96.3)	0.123

*.$\text{Traction Correction Rate }=\;\frac{\text{Post}-\text{Traction Cobb }-\text{ Initial Cobb}}{\text{Initial Cobb}}$

**.$\text{Surgical Correction Rate }=\;\frac{\text{Post}-\text{Operative Cobb }-\text{ Post}-\text{Traction Cobb}}{\text{Initial Cobb}}$

***.$\text{Total Correction Rate }=\;\frac{\text{Post}-\text{Operative Cobb }-\text{ Initial Cobb}}{\text{Initial Cobb}}$

The average follow up duration was 13 ± 1.2 months. There were no major complication associated with the use of preoperative traction in both groups. One patient has pin tract infection that was noticed at the end of traction period, treated with 5 days of intravenous antibiotics (ceftriaxone 2 × 1000 mg daily) and pin removal.

## 4 Discussion

The aim of this study is to evaluate and compare the safety and effectiveness of preoperative traction method (STFT and Cotrel) in treating severe and rigid scoliosis. Effectiveness of preoperative traction in severe scoliosis has been well recorded in previous studies.

Our study evaluated the effectiveness and safety of two preoperative traction method. Although both traction method yielded a good correction rate, there was no significant difference between them. However it should be noted that the STFT group had better post-traction and postoperative correction rate clinically. STFT procedure, although being more invasive, is hypothetically more effective than the less invasive Cotrel dynamic traction. The forces applied in the STFT group will be applied directly to the skeletal system which possibly explain the better correction rate obtained in the post-traction curve and postoperative curve. It should be noted that no vertebral column resection was performed in our series.

Preoperative traction was able to correct the deformity up to 23–35% during 3–4 weeks of traction [[15], [16], [17]]. In 2008, Sponseller performed a multicenter study involving more than 2000 patients, to evaluate the use of traction in severe spinal deformity. The study found that there was no significant difference in the final curve correction between traction and non-traction group. However, the need of vertebral column resection was significantly higher in non traction group [17]. Similar findings were found by Garabekyan et al., in their series of 21 severe spinal deformity treated with preoperative halo-gravity traction, there was no patient that underwent vertebral column resection or spinal osteotomy [16].

Cotrel traction may also have some benefit as a method of preoperative traction in severe and rigid scoliosis despite being inferior than STFT. Ramsey et al. showed that the use of preoperative cotrel traction increase the curve flexibility therefore it may contribute to a better surgical correction rate [18]. Nachemson & Nordwall demonstrated that the usage of cotrel traction did not improve the final correction rate [11]. Bjeirkreim et al. also concluded that preoperative Cotrel traction did not improve the surgical curve correction [19]. However, both of their study involved adolescence idiopathic scoliosis patients with lesser curve (40–90° curve), which would not really benefit from preoperative traction.

The ideal traction duration is still controversial. There was a wide variability among previous studies, ranging from 2 to 21 weeks [17,20,21]. Watanabe et al. claimed perioperative traction permits most of the correction of the coronal deformity within the first week, and should be applied for at least 3 weeks to obtain maximum deformity correction before definitive spinal fusion is performed [21]. Similar result was obtained by Park et al. in their review of 20 pediatric scoliosis cases treated with halo-gravity traction. More than 90% correction was achieved in 3 weeks with maximal correction was obtained after an average of 42.6 days [22]. In their series of 29 severe spinal deformity cases, Nemani stated the curve correction was rapid initially and then plateaued after 63 days of traction [23].

Longer traction time is associated with higher risk of complications, especially with the use of halo-femoral traction and Cotrel traction since these methods require a period immobilization. Longer period of complete immobilization increases the risk of disuse osteoporosis and fracture. Thus, in previous studies, the duration of halo-femoral traction was shorter compared to the halo-gravity traction, ranging from 20 to 77 days [24]. Keeping these risks in mind, our protocol was to shorten the traction duration even further (ranging from 7 to 21 days). During this period, the major curve correction has already been obtained. Furthermore the aim of the preoperative traction was not to obtain the maximal curve correction since it can be obtained even further during the definitive surgical procedure. Reducing the soft tissue tension and curve flexibility are the major factors to obtain an optimal curve correction.

In terms of safety, there was no major complications in both groups. There was some major complication related with the use of STFT, including perforation of the skull, brain abscesses, and neurovascular damage [25]. However, its number is very low. More often, it was associated with minor and transient complications, such as pin tract infection, which can be managed by pin removal and antibiotic administration which occurred in one case during our study. The disadvantages of cotrel traction is the effectiveness depends largely on the motivation of the patient and his/her parents. Barry et al. showed that cotrel traction may be a useful adjuvant in conjunction with bracing to maintain the flexability of the spine and in preventing reabsorption of bone [12].

There are several limitation of this study. The sample size is relatively small therefore associated with low statistical power, which was caused by the limited number of cases and strict criteria. The second one is the retrospective nature of this study that prevented us from obtaining a similar baseline charactheristic in both groups.

## 5 Conclusion

Both preoperative traction methods were found safe and beneficial to reduce preoperative curve degree before definitive scoliosis correction surgery. Although, no statistical difference were found between two methods, STFT may provide better correction rate.

## Ethical approval

Institutional review board approval was obtained from Fatmawati General Hospital, Jakarta.

## Fundings

The study did not receive any fundings.

## Author contribution

All authors contributed equally in study concept or design, data collection, data analysis and, writing the paper.

## Registration of research studies

1. Name of the registry: clinicaltrials.gov.

2. Unique Identifying number or registration ID: NCT04671147.

3. Hyperlink to your specific registration (must be publicly accessible and will be checked): https://clinicaltrials.gov/ct2/show/NCT04671147.

## Guarantor

Didik Librianto.

## Consent

The need for informed consent was waived due to the retrospective nature of the study. However the consent for publishing clinical and radiological images has been obtained.

## Provenance and peer review

Not commissioned, externally peer-reviewed.

## Declaration of competing interest

None.

## References

[1] Janicki J.A., Alman B. (2007). Scoliosis: review of diagnosis and treatment. Paediatr. Child Health.

[2] Little J.P., Izatt M.T., Labrom R.D. (2012). Investigating the change in three dimensional deformity for idiopathic scoliosis using axially loaded MRI. Clin Biomech (Bristol, Avon).

[3] committee Sg, Weiss H.R., Negrini S. (2006). Indications for conservative management of scoliosis (guidelines). Scoliosis.

[4] Mehrpour S., Sorbi R., Rezaei R. (2017). Posterior-only surgery with preoperative skeletal traction for management of severe scoliosis. Arch. Orthop. Trauma Surg..

[5] Edgar M.A., Chapman R.H., Glasgow M.M. (1982). Pre-operative correction in adolescent idiopathic scoliosis. J Bone Joint Surg Br.

[6] O'Brien J.P., Yau A.C., Hodgson A.R. (1973). Halo pelvic traction: a technic for severe spinal deformities. Clin. Orthop. Relat. Res..

[7] Stagnara P. (1971). [Cranial traction using the "halo" of rancho Los Amigos]. Rev Chir Orthop Reparatrice Appar Mot.

[8] Lerman J.A., Haynes R.J., Koeneman E.J. (1994). A biomechanical comparison of Gardner-Wells tongs and halo device used for cervical spine traction. Spine (Phila Pa 1976).

[9] Letts R.M., Palakar G., Bobecko W.P. (1975). Preoperative skeletal traction in scoliosis. J Bone Joint Surg Am.

[10] Dickson R.A., Leatherman K.D. (1978). Cotrel traction, exercises, casting in the treatment of idiopathic scoliosis. A pilot study and prospective randomized controlled clinical trial. Acta Orthop. Scand..

[11] Nachemson A., Nordwall A. (1977). Effectiveness of preoperative Cotrel traction for correction of idiopathic scoliosis. J Bone Joint Surg Am.

[12] Barry O.C., McManus F., Walshe M. (1983). Short term results of Cotrel traction in the treatment of idiopathic scoliosis. Ir. J. Med. Sci..

[13] Agha R., Abdall-Razak A., Crossley E. (2019). STROCSS 2019 Guideline: strengthening the reporting of cohort studies in surgery. Int. J. Surg..

[14] Rimel R.W., Butler A.B., Winn H.R. (1981). Modified skull tongs for cervical traction. Technical note. J Neurosurg..

[15] Bogunovic L., Lenke L.G., Bridwell K.H. (2013). Preoperative halo-gravity traction for severe pediatric spinal deformity: complications, radiographic correction and changes in pulmonary function. Spine Deform.

[16] Garabekyan T., Hosseinzadeh P., Iwinski H.J. (2014). The results of preoperative halo-gravity traction in children with severe spinal deformity. J. Pediatr. Orthop. B.

[17] Sponseller P.D., Takenaga R.K., Newton P. (2008). The use of traction in the treatment of severe spinal deformity. Spine (Phila Pa 1976).

[18] Ramsey P.L., Wickersham J., Kingsbury H. (1976). Mechanical analysis of Cotrel traction in idiopathic scoliosis. J Bone Joint Surg Am.

[19] Bjerkreim I., Carlsen B., Korsell E. (1982). Preoperative Cotrel traction in idiopathic scoliosis. Acta Orthop. Scand..

[20] Sink E.L., Karol L.A., Sanders J. (2001). Efficacy of perioperative halo-gravity traction in the treatment of severe scoliosis in children. J. Pediatr. Orthop..

[21] Watanabe K., Lenke L.G., Bridwell K.H. (2010). Efficacy of perioperative halo-gravity traction for treatment of severe scoliosis (>/=100 degrees). J. Orthop. Sci..

[22] Park D.K., Braaksma B., Hammerberg K.W. (2013). The efficacy of preoperative halo-gravity traction in pediatric spinal deformity the effect of traction duration. J. Spinal Disord. Tech..

[23] Nemani V.M., Kim H.J., Bjerke-Kroll B.T. (2015). Preoperative halo-gravity traction for severe spinal deformities at an SRS-GOP site in West Africa: protocols, complications, and results. Spine (Phila Pa 1976).

[24] Bonnett C., Brown J.C., Perry J. (1975). Evolution of treatment of paralytic scoliosis at rancho Los Amigos hospital. J Bone Joint Surg Am.

[25] Hresko M.T. (2013). Clinical practice. Idiopathic scoliosis in adolescents. N. Engl. J. Med..

